# Integrated Transcriptomic and Metabolomic Analyses Reveal Key Responses of Cotton to Salt Stress Post-Germination

**DOI:** 10.3390/cimb47110951

**Published:** 2025-11-15

**Authors:** Yanzhen Liu, Yaxin Shi, Ren Xiang, Jianduo Bai, Jingshun Wang, Xianliang Zhang

**Affiliations:** 1 College of Biology and Food Engineering, Anyang Institute of Technology, Anyang 455000, China; 13783858251@163.com (Y.L.); renxiang@caas.cn (R.X.); aywjs8@163.com (J.W.); 2 Western Research Institute, Chinese Academy of Agricultural Sciences (CAAS), Changji 831100, China; bjdzky@163.com; 3 School of Horticulture and Landscape Architecture, Henan Institute of Science and Technology, Xinxiang 453003, China; 17639846747@163.com

**Keywords:** cotton, salt stress, metabolomics, transcriptome, seed germination

## Abstract

Salt stress is a major environmental constraint that severely limits cotton seed germination. However, the regulatory mechanisms governing salt stress responses during the post-germination stage remain largely unclear. Here, we employed an integrated transcriptomic and metabolomic approach to investigate salt-responsive mechanisms in the salt-tolerant cotton cultivar ST022-1056m5 (ST) following exposure to 150 mM NaCl. Our analysis identified 4368 differentially expressed genes (DEGs) and 441 differentially accumulated metabolites (DAMs) under salt stress conditions. Multi-omics integration revealed that alpha-linolenic acid and linoleic acid metabolic pathways were particularly responsive to salt stress. In the α-linolenic acid pathway, salt stress triggered substantial accumulation of jasmonic acid (JA) precursors and concurrent upregulation of key JA biosynthetic genes. Simultaneously, the linoleic acid metabolism pathway exhibited increased metabolite levels and enhanced the relative gene expression. These findings provide compelling evidence that alpha-linolenic acid and linoleic acid metabolism pathways collectively modulate post-germination salt stress responses in cotton, offering new insights into the molecular mechanisms underlying salt tolerance and presenting potential targets for breeding resilient cotton varieties.

## 1. Introduction

Cotton, a major cash crop of the Malvaceae family, is cultivated worldwide because of its high-quality fiber and valuable oilseed [1]. Moreover, population growth worldwide has increased the demand for cotton, driving the continued expansion of its cultivation. However, soil salinization has emerged as a significant constraint on agricultural productivity, affecting over 20% of irrigated cropland across at least 75 countries [2,3]. This issue is further exacerbated by climate change and anthropogenic activities. Salinized land is reportedly increasing by approximately 1 million hectares annually [4], and by 2050, nearly 50% of agricultural land is projected to experience varying degrees of salt damage [5]. In China alone, an estimated 99 million ha of salinized land has been identified in Xinjiang, the country’s leading cotton-producing region, thus posing a substantial challenge [6]. Beyond reducing cotton yield and quality, soil salinization impedes rural development. Therefore, breeding salt-tolerant cotton cultivars has become an economically and agronomically viable strategy for utilizing marginal land.

Although cotton exhibits a degree of salt tolerance, its germination and emergence till formation of first reproductive branches are vulnerable to high saline conditions (more than 7.7 dS m^−1^), leading to reduced yield and poorer fiber quality [7]. Seed germination typically occurs in three sequential phases [8,9]. Phase I (imbibition) involves rapid water uptake and hydration of macromolecules, leading to reactivation of mitochondrial respiration and DNA/membrane repair systems. Phase II (metabolic activation) is marked by the de novo synthesis of RNA, lipids, and proteins, alongside the mobilization of stored nutrients, thereby enabling the transition from dormancy to germination. Phase III (post-germination) is characterized by radicle emergence driven by cell division and elongation in the plumular axis, thereby promoting seedling establishment and causing a loss of desiccation tolerance [10,11]. Due to the structural irreversibility of this stage, cotton is particularly vulnerable to salt stress during germination [7,12]. While numerous studies have elucidated salt-responsive genes and pathways in cotton leaves, roots, fibers, and seeds during early germination [13,14,15], the post-germination stage remains underexplored.

Plants respond to environmental stress through dynamic metabolic reprogramming, which generates metabolites that serve both adaptive functions and potential biomarkers [16]. Metabolomic analyses have identified key metabolites that contribute to salt stress tolerance across several crops [17,18]. For example, soybean accumulates catechin, L-homocysteine, and kaempferol to mitigate osmotic stress under saline-alkaline conditions [19]. In grapevine, salt stress induces the metabolism of nitrogen and increases the accumulation of free amino acids, such as glutamate, proline, and polyamine putrescine, and the non-protein amino acid γ-aminobutyrate [20]. In wheat seedlings, salinity triggers the accumulation of flavonoids, organic and phenolic acids, amino acids and their derivatives, and phytohormones [21]. These findings underscore the utility of metabolomics in elucidating plant stress adaptations and mapping metabolic networks, particularly at critical developmental stages such as post-germination.

Salt tolerance in plants is orchestrated by complex gene regulatory networks [22]. Although salinity induces conserved patterns of metabolic reprogramming [23], the responses exhibit marked spatial, temporal, and varietal specificity [24]. Consequently, transcriptomic or metabolomic analysis alone is insufficient for fully characterizing salt tolerance mechanisms. Integrating transcriptomic and metabolomic datasets enables systematic identification of key stress-responsive genes, core metabolic pathways, and their regulatory interactions. This approach not only elucidates mechanisms of salt tolerance but also informs molecular breeding strategies [25]. Association analysis of transcriptome and metabolome data has thus emerged as a powerful tool for dissecting the molecular basis of salt tolerance and accelerating the development of stress-resilient cultivars [26,27].

In this study, we employed an integrated transcriptomic–metabolomic approach to investigate the molecular response of the salt-tolerant cotton cultivar ST022-1056m5 to salt stress during the post-germination stage. This combined analysis identified key salt-responsive metabolic pathways and candidate regulatory genes, thus providing comprehensive insights into the molecular basis of early-stage salinity tolerance in cotton and supporting the development of salt-tolerant cultivars.

## 2. Materials and Methods

### 2.1. Plant Materials and Experimental Design

The salinity tolerance of 47 cotton cultivars with contrasting performance was determined through field assays (Table S2). From these, the salt-tolerant cotton cultivar ST022-1056m5 (ST, salt-tolerant) and the salt-sensitive cultivar ZM113 (SS) were used as experimental material. The experimental procedure was adapted from Zhang et al. [28] with minor modifications. Briefly, uniform full seeds were surface-sterilized with 10% (*v*/*v*) hydrogen peroxide for 30 min, followed by four to five rinses with sterile distilled water. After blotting dry with sterile filter paper, seeds from each genotype were randomly divided into two groups: 150 mM NaCl treatment group and the water control group. Six independent biological replicates of 30 seeds each were prepared per treatment. Three replicates were germinated in a chamber (28 °C, 14/10 h light/dark) for seven days to record daily germination (defined as radicle emergence ≥ 2 mm) and to measure primary root length using ImageJ version 2.0. The remaining three replicates were planted in a peat-based compost mixture and subjected to either a control (water) or a 150 mM NaCl treatment in a growth chamber under the same conditions; seedling scoring was conducted after five and seven days of growth.

### 2.2. LC-MS/MS Analysis

Following three days of treatment with either salt or water, a biological replicate consisting of 10 germinated ST seeds was collected and homogenized into a powder using liquid nitrogen. Three biological replicates were included for each treatment. Metabolite extraction was performed using grinding beads, and LC-MS/MS analysis was conducted by Majorbio Bio-Pharm Technology Co., Ltd. (Shanghai, China) [29].

### 2.3. Metabolomic Analysis

The resulting data matrix was analyzed on the Majorbio Cloud Platform (https://cloud.majorbio.com, accessed on 20 May 2025). Principal component analysis (PCA) and orthogonal partial least squares discriminant analysis (OPLS-DA) were used for multivariate analysis. Metabolites with variable importance in projection (VIP) scores > 1 and *p* < 0.05 (based on Student’s *t*-test) were defined as significantly different [30]. Differential metabolites were subjected to KEGG pathway enrichment analysis (http://www.genome.jp/kegg/) and mapped to relevant metabolic pathways [31]. Metabolites were categorized according to associated pathways and biological functions.

### 2.4. RNA-Seq Analysis

Total RNA was extracted from the powder for metabolomic analysis, with three biological replicates performed per treatment. Strand-specific RNA-seq libraries were constructed on the Illumina platform. After removal of rRNA, the target RNA fragments were enriched, followed by second-strand cDNA synthesis, adapter ligation, enrichment, and sequencing [32]. Clean reads were aligned to the *Gossypium hirsutum* TM-1 reference genome (http://cotton.zju.edu.cn/source/TM-1_V2.1.fa.gz, accessed on 20 May 2025) using HISAT2 v2.0.5 [33]. Differentially expressed genes (DEGs) were identified using DESeq2 with an adjusted *p*-value < 0.01 and |log_2_ fold change| ≥ 1 [34].

### 2.5. Integrated Transcriptomic and Metabolomic Analysis

DEGs and differentially expressed metabolites (DEMs) were visualized using iPath 3.0 (https://pathways.embl.de) [35]. To explore metabolite–gene correlations, eight representative DEMs (VIP > 1, log_2_FC > 1, *p* < 0.05) and corresponding significant DEGs (log_2_FC > 1, *p* < 0.05, FPKM > 10) were selected for co-expression network analysis using Pearson correlation (r > 0.90). Network construction and visualization were performed in Cytoscape version 3.9, and KEGG Mapper (https://www.kegg.jp/kegg/mapper.html, accessed on 20 May 2025) was used for pathway annotation.

### 2.6. qRT-PCR Validation

TRNA-seq results were validated by quantitative real-time PCR (qRT-PCR) following the method of Zhang et al. [36]. Three biological replicates were used per treatment. First-strand cDNA was synthesized from total RNA using the HiScript III 1st Strand cDNA Synthesis Kit with gDNA wiper (Vazyme, Nanjing, China). Specific primers were designed for nine DEGs (Table S1) and the reference gene *GhActin9* [37]. qRT-PCR was performed on a Bio-Rad iQ5 system (Hercules, CA, USA) using SYBR Green PCR SuperMix (Vazyme, China). Relative gene expression was calculated using the 2^−ΔΔCt^ method.

### 2.7. Data Processing, Visualization, and Statistical Analysis

Bar graphs were generated using GraphPad Prism 6.0, and heat maps of gene expression (FPKM values) were produced in TBtools v2.025 [38]. Statistical significance was assessed using the least significant difference (LSD) method at *p* < 0.05 or *p* < 0.01. All experiments included at least three biological replicates.

## 3. Results

### 3.1. Salt Tolerance Assay of Cotton Cultivars During Germination and Post-Germination Stages

**Figure 1:**
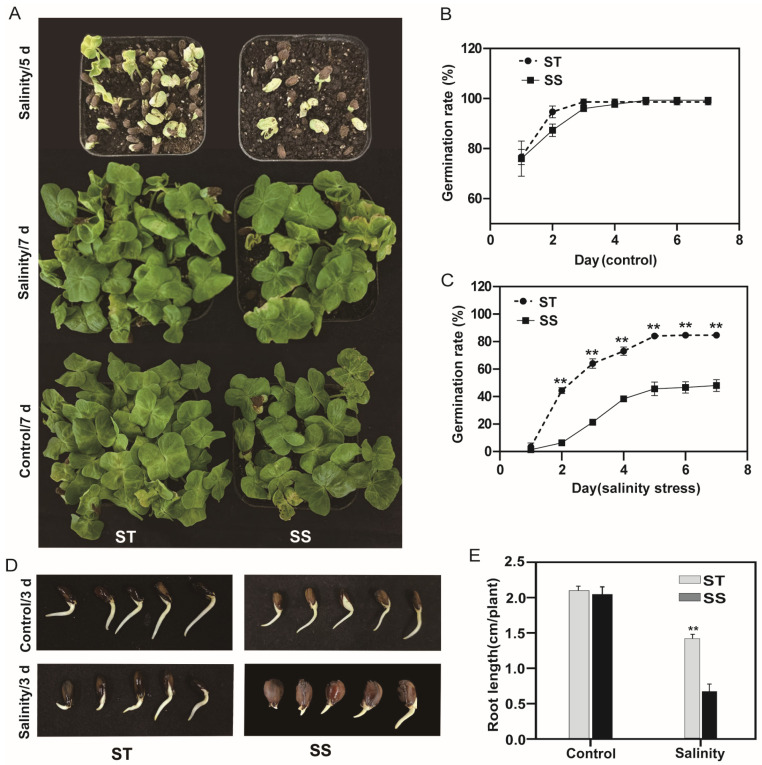
Analysis of salinity tolerance during germination and early seedling growth. (**A**) Germination under control (water) or salt (150 mM NaCl) conditions over time. (**B**) Germination percentage under control conditions. (**C**) Germination percentage under salt treatment. (**D**) Primary root growth under control and salt conditions after 3 days. (**E**) Root length under control or salt treatment after three days. Data represent means ± SD (*n* = 30 seeds). Asterisks (**) indicate significant differences (*p* < 0.01; *t*-test). All experiments were performed in triplicate.

To investigate the mechanisms of salinity tolerance in *G. hirsutum*, we selected the ST022-1056m5 ST and ZM113 SS lines from 47 cultivars exhibiting contrasting salinity tolerance, as determined by field assays (Table S2). Under control conditions, ST and SS showed comparable germination rates and reached the post-germination stage (Figure 1). In contrast, under salt stress, ST exhibited markedly enhanced performance, more than 80% of seeds germinating after 5 days of treatment compared to 53% for SS (Figure 1A–C), confirming ST as a salt-tolerant cultivar. This demonstrates that ST maintained high germination efficiency (>80%) under both control and saline conditions. Furthermore, most ST seeds showed rapid radicle elongation, reaching 1.5 cm by the third day (Figure 1D,E), indicating that ST seeds had progressed to the post-germination stage by this time point.

### 3.2. Metabolomic Characteristics of ST in the Post-Germination Stage Under Salt Stress

**Figure 2:**
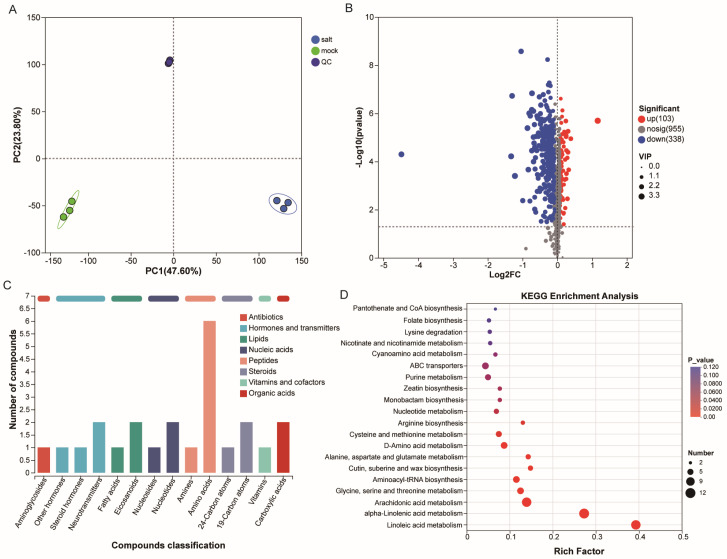
Metabolomic profiling of ST post-germination stages in response to exogenous salt stress. (**A**) Principal component analysis (PCA) of metabolites in control- and salt-treated samples. (**B**) Volcano plot of differentially expressed metabolites (DEMs). (**C**) Classification of DEMs by KEGG category. (**D**) KEGG pathway enrichment analysis of DEMs.

To characterize metabolic responses in ST in the post-germination stage under salt stress, LC-MS-based broad-target metabolomics was performed on samples collected on day 2 of treatment. PCA revealed distinct separation between the metabolic profiles of the salt-treated and control samples (Figure 2A), with PC1 and PC2 explaining 47.60% and 23.80% of the variance, respectively. Partial least squares discriminant analysis (PLS-DA) further confirmed the separation between treatment groups (Figure S1). Based on the criteria *p* < 0.05 and VIP > 1, a total of 441 DEMs were identified (Figure 2B), including 103 upregulated and 338 downregulated metabolites. KEGG classification revealed that these DEMs were predominantly enriched in the “hormones and transmitters” category (Figure 2C). Among them, nicotinic acid ribonucleoside exhibited a high VIP score and strong differential expression, suggesting a key role for carbohydrates and their derivatives in the salt stress response. Hierarchical clustering analysis indicated that approximately two-thirds of the DEMs were downregulated in salt-treated seedlings, forming ten distinct metabolite clusters (Figure S2).

Key metabolic alterations under salt stress were further analyzed through KEGG pathway enrichment (Figure 2D, Table S3). The significantly enriched pathways included “linoleic acid metabolism”, “cutin, suberine and wax biosynthesis”, “arachidonic acid metabolism”, “glycine, serine and threonine metabolism”, “aminoacyl-tRNA biosynthesis”, and “alpha-linolenic acid metabolism.” These findings suggest that exogenous salt stress modulates both carbohydrate and lipid metabolic pathways, thereby contributing to the salt stress response in cotton seedlings.

### 3.3. Identification of Salt Stress-Responsive Genes in ST Post-Germination Stage by Transcriptome Analysis

**Figure 3:**
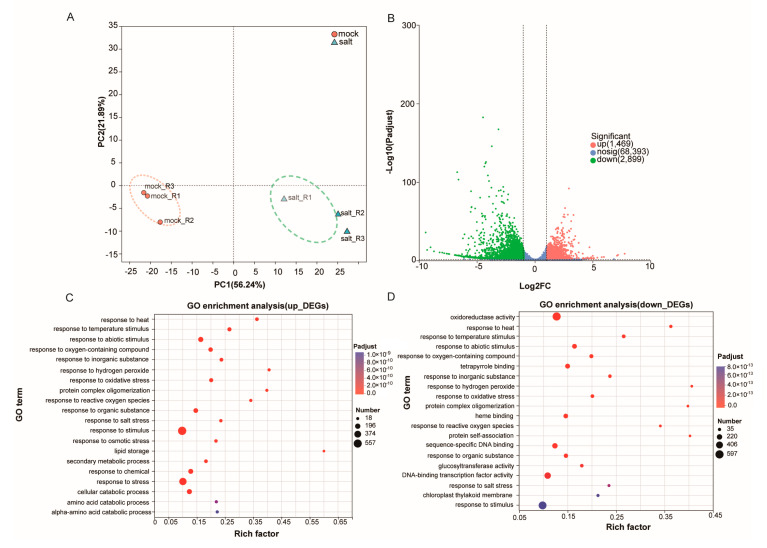
Transcriptomic profiling of ST in the post-germination stage under exogenous salt stress. (**A**) PCA of transcriptomic profiles of the control and salt-treated samples. (**B**) Volcano plot showing differentially expressed genes (DEGs) between control and salt-treated seedlings; red and blue dots represent significantly up- and downregulated genes, respectively. (**C**,**D**) Gene Ontology–Biological Process (GO-BP) enrichment analysis of (**C**) upregulated and (**D**) downregulated DEGs; top enriched terms shown (FDR < 0.05).

To systematically identify salt stress-responsive genes in the ST post-germination stage, RNA-seq analysis was conducted on samples collected on the third day of treatment. PCA revealed clear separation between salt-treated and control samples along PC1, which accounted for 56.24% of the total variance, while PC2 accounted for 21.89% (Figure 3A). Differential expression analysis identified 4368 DEGs (|log_2_FC| > 1; adjusted *p* < 0.05), indicating extensive transcriptional reprogramming under salt stress (Figure 3B).

Gene Ontology–Biological Process (GO-BP) enrichment analysis showed that these DEGs were significantly enriched in terms such as “response to salt stress”, “response to stimulus”, “response to osmotic stress” and “response to abscisic acid” (Table S4), suggesting the activation of multiple stress- and hormone-related pathways in ST under salt stress. Further GO-BP enrichment of upregulated and downregulated DEGs revealed distinct functional patterns (Figure 3C,D). Upregulated genes were enriched in terms such as “response to abiotic stimulus”, “response to salt stress”, “response to osmotic stress”, “secondary metabolic process” and “lipid storage” with particularly strong enrichment in general stress-responsive terms. In contrast, downregulated genes were enriched in “response to oxidative stress”, “oxidoreductase activity” and “response to inorganic substance.” These findings suggest that ST seedlings undergo multilevel transcriptional reprogramming during early salt stress by simultaneously activating protective pathways and repressing select metabolic processes to enhance stress adaptation.

### 3.4. Integrated Metabolome–Transcriptome Analysis

**Figure 4:**
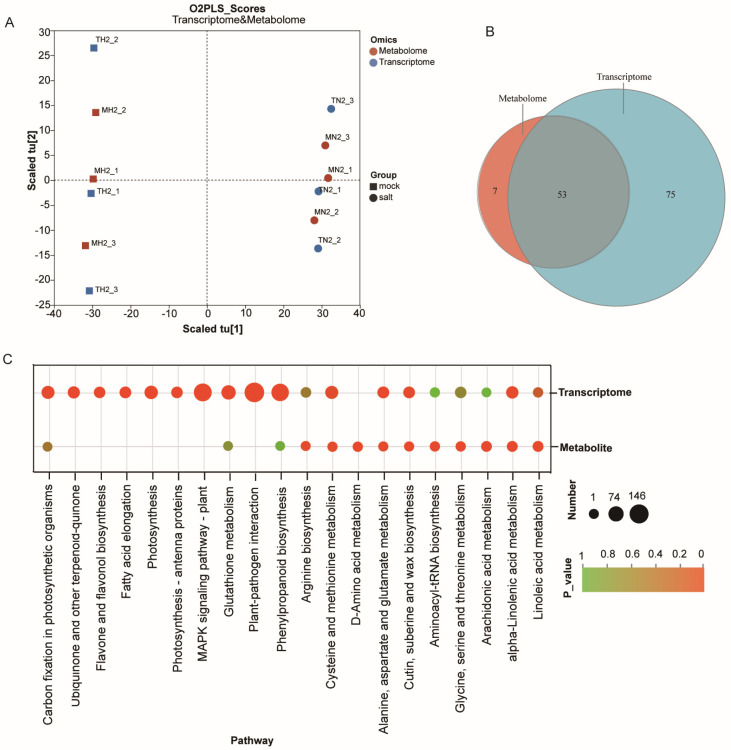
Correlation between the metabolomic and transcriptomic responses to salt stress. (**A**) O2PLS score plot showing joint variation between metabolomic and transcriptomic datasets; T-values represent transcriptomic scores, and U-values represent metabolomic scores. (**B**) Venn diagram of KEGG pathways enriched in DEGs and DEMs. (**C**) Top 20 KEGG pathways containing the highest number of DEGs and DEMs identified by integrated omics analysis.

To further explore the relationship between salt-responsive metabolic and transcriptional changes in ST post-germination stage under salt stress, we performed an integrated analysis of DEGs and DEMs using an O2PLS model. A high degree of correlation was observed between the two omics datasets, indicating coordinated regulation (Figure 4A). Venn diagram analysis showed substantial overlap between the KEGG pathways enriched in DEMs and DEGs (Figure 4B,C). Co-enriched pathways included “linoleic acid metabolism”, “alpha-linolenic acid metabolism”, “alanine, aspartate, and glutamate metabolism” and “glycine, serine, and threonine metabolism”. Given that metabolite accumulation levels were influenced by corresponding gene expression changes, this suggests a strong coupling between transcriptional regulation and metabolic remodeling under salt stress.

Given that metabolite accumulation is often regulated at the transcriptional level, we conducted a correlation analysis of the transcriptomic and metabolomic datasets. iPath-based pathway mapping indicated that DEGs and DEMs jointly contributed to key biological processes, including “amino acid metabolism”, “biosynthesis of other secondary metabolites”, “carbohydrate metabolism” and “lipid metabolism” (Figure S3A). To identify regulatory hubs, we selected eight core metabolites from the top 8 DEMs to construct a co-expression network with 50 major DEGs (Figure S3B). The resulting network revealed distinct regulatory clusters, with several DEMs exhibiting strong co-expression with multiple DEGs. These results suggest that specific genes may directly or indirectly regulate the biosynthesis and accumulation of critical metabolites involved in the salt stress response.

### 3.5. Integrated Analysis of Genes and Metabolites Related to Alpha-Linolenic Acid and Linoleic Acid Metabolism in ST in the Post-Germination Stage Under Salt Stress

**Figure 5:**
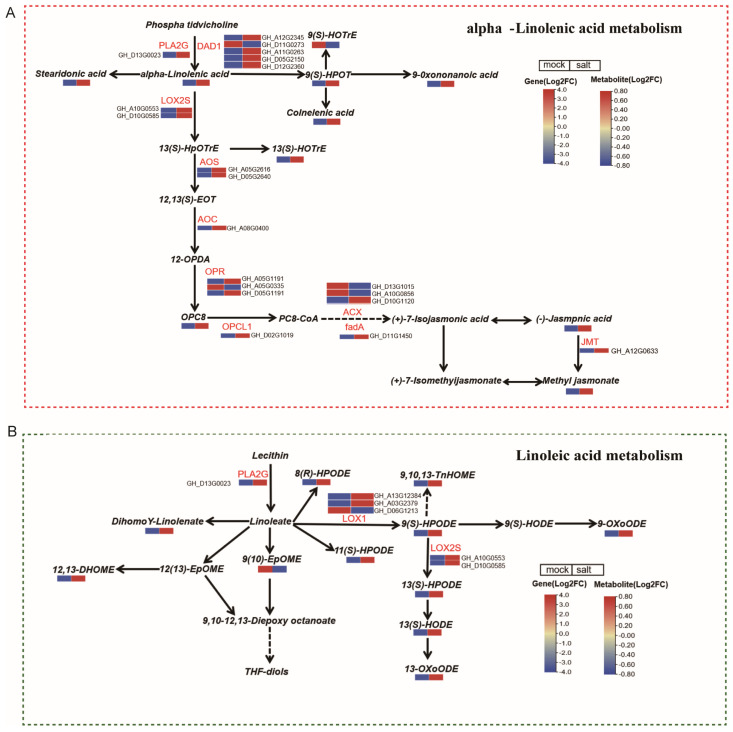
DEGs and DEMs involved in (**A**) alpha-linolenic acid metabolism and (**B**) linoleic acid metabolism in early-stage cotton seedlings under salt stress. Each rectangular block is split in half, representing expression under salt (**left**) and control (**right**) conditions for DEGs and differentially accumulated metabolites (DAMs).

Key regulators of the alpha-linolenic acid metabolic pathway in the early-stage ST seedlings under salt stress were identified through an integrated analysis of DEGs and DEMs (Figure 5A, Tables S5 and S6). This pathway exhibited significant alterations at both the transcriptomic and metabolomic levels, with 20 DEGs identified. Notably, most genes involved in alpha-linolenic acid metabolism were upregulated, although four enzyme-coding genes were downregulated.

Specifically, genes encoding 12-oxophytodienoic acid reductase (*OPR*; *GH_D05G1191*, *GH_A05G1191*), lipoxygenase (*LOX*; *GH_D10G0585*, *GH_A10G0553*), and HRAS-like suppressor 3 (*DAD1*; *GH_D12G2360*, *GH_A11G0263*) were upregulated by 10- to 86.43-fold. Metabolomic profiling revealed that jasmonic acid levels increased by more than 1.6-fold under salt stress, and colnelenic acid, 9-oxononanoic acid, 13(S)-HOTrE, 13(S)-HpOTrE, and OPC8 levels were also increased. These findings support a synergistic regulatory relationship between transcriptomic and metabolomic changes within the alpha-linolenic acid metabolism pathway.

To further explore linoleic acid metabolism, an interaction network was constructed that integrated DEGs and DEMs associated with this pathway (Figure 5B; Tables S5 and S6). Linoleic acid metabolism was also significantly reprogrammed in response to salt stress, with six DEGs identified, five upregulated and one downregulated. The gene encoding secretory phospholipase A2 (*PLA2G*; *GH_D13G0023*) was upregulated, while four lipoxygenase genes (*LOX1*; *GH_A06G1213*) were downregulated. Conversely, lipoxygenase genes (*LOX2; GH_A10G0553*, *GH_D10G0585*) were significantly upregulated by 12.12- and 23.04-fold, respectively. Metabolite analysis revealed substantial increases in 11-HpODE, 12,13-DiHOME, dihomo-γ-linolenic acid, coriolic acid, and 9,10,13-TriHOME. These results further confirm that linoleic acid metabolism responds to salt stress via coordinated changes in gene expression and metabolite accumulation.

### 3.6. qRT-PCR Validation of DEGs in ST in the Post-Germination Stage Under Salt Stress

**Figure 6:**
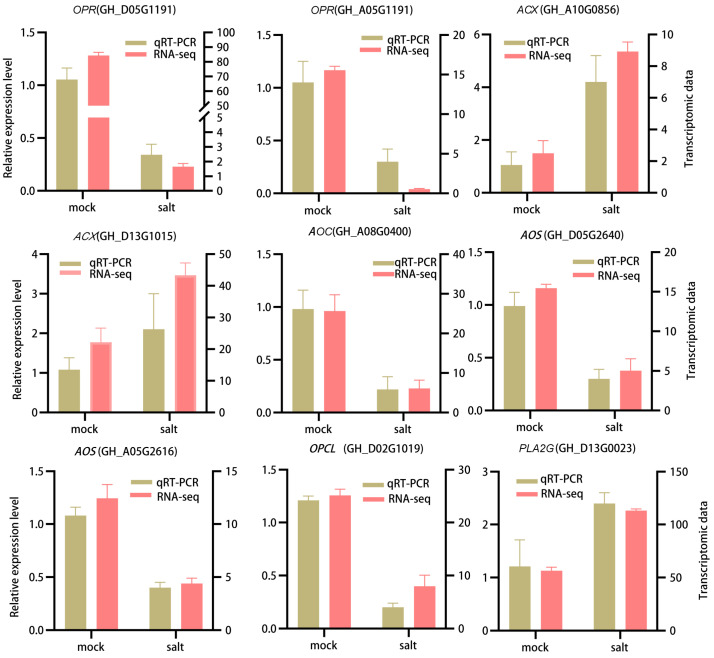
qRT-PCR validation of selected DEGs. Gene abbreviations: *OPR*, 12-oxophytodienoic acid reductase; *ACX*, acyl-CoA oxidase; *AOC*, allene oxide cyclase; *AOS*, hydroperoxide dehydratase; *OPCL*, OPC-8:0 CoA ligase, *PLA2G*, HRAS-like suppressor 3.

To validate the reliability of the transcriptomic data, qRT-PCR was performed on eight representative DEGs involved in alpha-linolenic acid, including *OPR*, *ACX*, *AOC*, *AOS*, and *OPC*. As shown in Figure 6, the expression patterns observed by qRT-PCR were consistent with the transcriptomic data, demonstrating similar trends of upregulation under salt stress. This concordance confirms the robustness and reproducibility of the RNA-seq results.

## 4. Discussion

Soil salinity is a significant limiting factor for crop growth, yield, and production, is becoming a severe threat to most crop plants [39]. Although cotton exhibits relatively strong salt tolerance compared to other crops, its sensitivity varies significantly across developmental stages. The seed germination and early seedling stages are particularly vulnerable because high NaCl concentrations inhibit root growth while promoting excessive uptake and translocation of toxic Na^+^ and Cl^−^ to shoots. This ionic imbalance disrupts cellular homeostasis, leading to stunted seedling establishment and reduced crop. These physiological disruptions often lead to poor stand establishment and ultimately reduce crop yield [40]. Therefore, the key responses and related mechanisms involved in seed germination and post-germination under salt stress must be determined. Moreover, investigating the salt-responsive genes or metabolites in the post-germination stage under salt stress can expand our current knowledge of cotton salinity tolerance and identify useful genes for improving tolerance during the cotton breeding program. In this study, we found that the salt-tolerant cotton cultivar ST exhibited vigorous root growth, with root length exceeding twice the seed length (≥2-fold) after three days under both control (water) and salt-stress conditions (Figure 1). This rapid root elongation, which aligns with phenotyping criteria for cotton germination studies [28,41,42], clearly indicates the successful transition to the early seedling stage.

Transcriptomic analysis has become a powerful approach for characterizing salt-stress responsive genes and elucidating their molecular functions during cotton seed germination and early seedling development [43,44,45,46]. In this study, comparative transcriptome profiling of the salt-tolerant cotton cultivar ST under salt stress revealed 4368 differentially expressed genes (DEGs) relative to the water-treated control. GO enrichment analysis classified these DEGs into several key functional categories (Figure 3, Table S4), such as response to abiotic stimulus (GO:0009628), response to salt stress (GO:0009651), response to osmotic stress (GO:0006970), response to abscisic acid (GO:0097305), response to water (GO:0009415), response to hormone (GO:0006520), cell wall biogenesis (GO:0009833), and lipid metabolic process (GO:0006629). Notably, the functional categories of these salt-responsive genes showed significant consistency with previous transcriptomic studies for cotton seed germination under salt stress [33,47,48]. These robust findings not only validate our experimental approach but also provide a reliable foundation for subsequent metabolomic correlation analyses to further dissect the molecular mechanisms of salt tolerance in cotton.

Plant metabolism plays a pivotal role in stress resistance, with the accumulation of specialized metabolites such as flavonoids, terpenes, lipids, and hormones being well-documented as key mediators of stress adaptation and tolerance across diverse plant species [49,50,51]. In this study, we observed significant activation of nicotinate and nicotinamide metabolism in the salt-treated plants during early post-germination. This metabolic shift resulted in the marked accumulation of two key compounds: nicotinic acid ribonucleoside (which exhibited the highest VIP value; Figure 3, Table S2) and niacinamide. Similar increases in niacinamide content have been reported in salt-stressed tobacco [52], rapeseed [53], and water lettuce [54], suggesting a conserved stress-response mechanism among divergent species. Functionally, nicotinamide serves as a vital component of the pyridine dinucleotide coenzyme NAD(P)H, which acts as both a stress signal and a critical mediator in enzymatic oxidation-reduction reactions [55]. Other studies have demonstrated that nicotinamide enhances carbohydrate production and protein synthesis in sorghum under salt stress [56]. Collectively, these findings suggest that the observed upregulation of nicotinate and nicotinamide metabolism may be crucial for sustaining the alpha-linolenic acid metabolism and linoleic acid metabolism that were observed in our enriched pathways (Figure 4), which help cotton maintain osmotic and metabolic homeostasis during stress conditions.

Integrative analysis of the transcriptomes and metabolomes showed that DEGs and DEMs were mostly associated with the “alpha-linolenic acid metabolism”, “linoleic acid metabolism”, “alanine, aspartate and glutamate metabolism” and “glycine, serine and threonine metabolism” pathways (Figure 4). The alpha-linolenic acid metabolism pathway begins with the peroxidization of alpha-linolenic acid by lipoxygenase. Subsequently, under the influence of lipoxygenase (LOX) genes, alpha-linolenic acid is converted into 13-hydroperoxy-9,11,15-octadecatrienoic acid (13-HPOT), the product of which is then converted to 12,13(S)-epoxy-octadecatrienoic acid (12,13-EOT) by the enzyme allene oxide synthase (AOS). 12,13-EOT is then processed to 12-oxo-phytodienoic acid (OPDA) by the action of allene oxide cyclase (AOC). Afterward, OPRII reduces cis-(+)-OPDA to form 3-oxo-2-(29-[Z]-pentenyl) cyclopentane-1 octanoic acid (OPC 8:0), which is finally oxidized to produce JA by acyl-CoA oxidase (ACX) [57,58]. Numerous studies have shown that alpha-linolenic acid can not only act as a strong antioxidant but also as a precursor to the synthesis of JA, which acts as a signaling molecule to stimulate the downstream anti-stress response [59,60]. In this study, alpha-linolenic acid and jasmonic acid in the alpha-metabolism pathway were significantly accumulated (Figure 5A), and some structural genes in this pathway were also significantly upregulated, such as *PLA2G*(*GH_D13G0023*), *AOS*(*GH_D05G2640*), *OPR*(*GH_A05G1191* and *GH_D05G1191*), *ACX*(*GH_D10G1120*) and *JMT*(*GH_A12G0633*). These results highlight that alpha-linolenic acid metabolism is mostly involved in the synthesis of JA during the early seedling stage and alleviates salt stress through JA signaling. In addition, genes involved in the metabolism of alpha-linolenic acid have been extensively reported to be associated with stress resistance in plants [61]. For example, in rice, cold stress induces *OsAOS1*, *OsAOS2*, *OsOPR1*, and *OsOPR7* expression, thereby elevating endogenous JA levels [62]; in *Arabidopsis*, the cotton gene *GhACX3* can increase the seed germination rate and survival rate [63]; and in tomato, *SlPLA1-2* enhances cold tolerance by regulating CBF signaling and JA biosynthesis in coordination [64]. The identified upregulated genes from the alpha-linolenic acid pathway represent promising molecular targets for developing stress-resistant crop varieties through genetic engineering or marker-assisted breeding approaches.

Cottonseed oil is predominantly composed of linoleic acid (C18:2), which constitutes more than 50% of its total fatty acids, followed by palmitic acid (C16:0), oleic acid (C18:1), and stearic acid (C18:0) [65]. As a key constituent of triacylglycerols and glycerolipids, linoleic acid plays multiple physiological roles, including carbon and energy storage, regulation of membrane lipid fluidity, and serving as a precursor for various bioactive molecules [66]. The accumulation of linoleic acid has been widely reported in plants subjected to abiotic stresses such as low temperature, chilling, and salt stress [67,68]. In the present study, we observed that several metabolites in the linoleic acid metabolism pathway, including 11-HpODE, 12,13-DiHOME, dihomo-γ-linolenic acid, coriolic acid, and 9,10,13-TriHOME were significantly upregulated under salt treatment in the salt-tolerant cultivar ST. Furthermore, key genes involved in linoleic acid metabolism, such as the lipoxygenase genes *LOX2* (*GH_A10G0553*, *GH_D10G0585*), were also notably induced (Figure 5B). These findings further indicate that linoleic acid metabolism plays an important role in enhancing salt tolerance during the post-germination stage of the ST cultivar.

## 5. Conclusions

This study employed integrated transcriptomic and metabolomic analyses to elucidate the molecular mechanisms underlying cotton’s response to salt stress during the post-germination stage. A total of 4368 DEGs and 441 DAMs were identified. KEGG enrichment analysis revealed significant involvement of the alpha-linolenic acid and linoleic acid metabolism pathways under salt stress. Construction of a regulatory network integrating genes and metabolites from these pathways demonstrated strong correlations between the expression of key genes and the accumulation of associated metabolites. Notably, salt stress induced substantial accumulation of metabolites in both the α-linolenic acid and linoleic acid pathways, accompanied by the upregulation of key regulatory genes, such as *OPR*, *DAD1*, *LOX*, *AOC*, and *AOS*. These findings underscore the pivotal role of α-linolenic acid and linoleic acid metabolism in mediating salt tolerance during the post-germination stage.

## Data Availability

Clean RNA-Seq data have been submitted to the National Center Biotechnology Information (Accession ID PRINA1335884). The original contributions presented in this study are included in the article/Supplementary Materials. Further inquiries can be directed to the corresponding author.

## Supplementary Materials

The supporting information can be downloaded at: https://www.mdpi.com/article/10.3390/cimb47110951/s1.
