# Supplementary material for: Integrated Transcriptomic and Metabolomic Analyses Reveal Key Responses of Cotton to Salt Stress Post-Germination
Source: Curr Issues Mol Biol. 2025 Nov 15;47(11):951. doi: 10.3390/cimb47110951 (PMC12651791; doi:10.3390/cimb47110951)
Supplement: Supplementary file 1 [file cimb-47-00951-s001.zip › Figure S1.pdf]

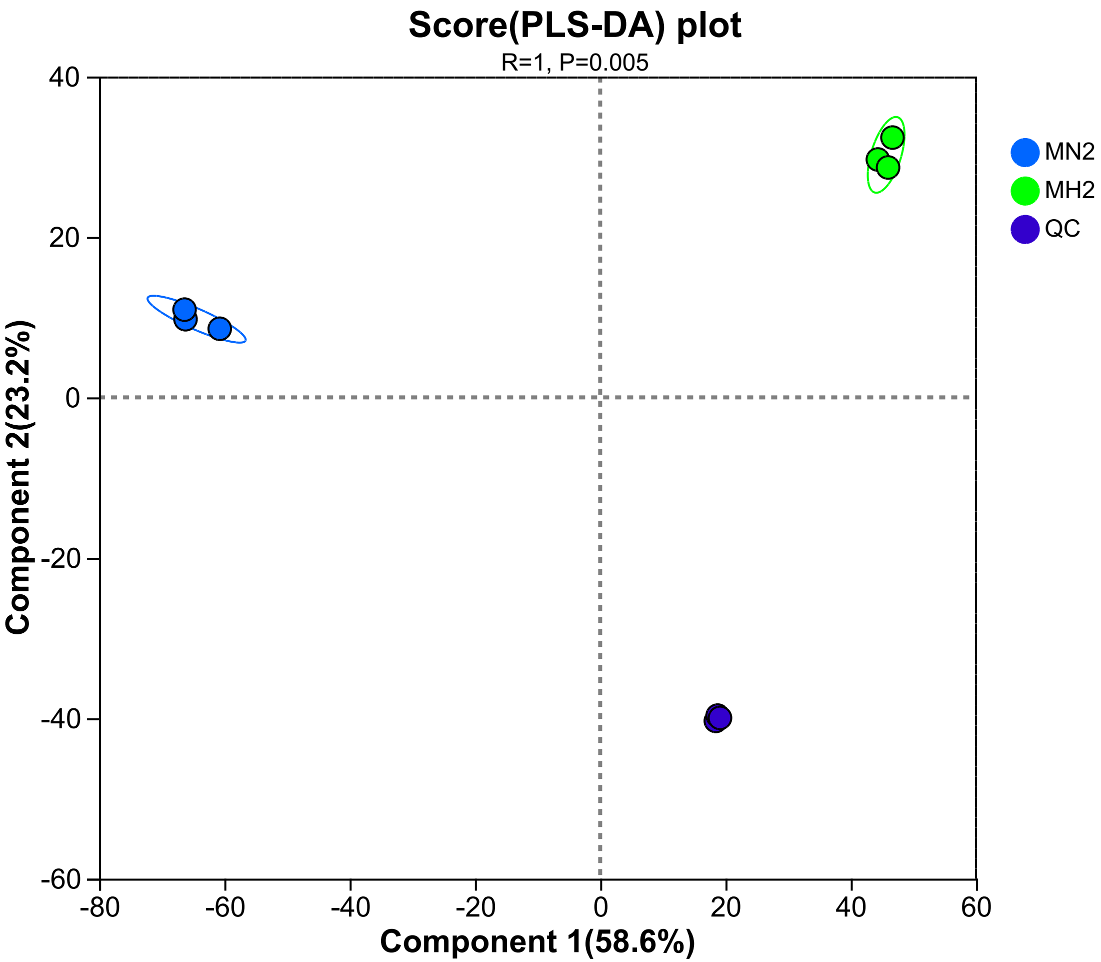

Figure S1: Partial least squares discriminant analysis (PLS-DA) score plot of metabolic profiles under salt and water treatments.
