# Supplementary material for: Integrated Transcriptomic and Metabolomic Analyses Reveal Key Responses of Cotton to Salt Stress Post-Germination
Source: Curr Issues Mol Biol. 2025 Nov 15;47(11):951. doi: 10.3390/cimb47110951 (PMC12651791; doi:10.3390/cimb47110951)
Supplement: Supplementary file 1 [file cimb-47-00951-s001.zip › Figure S2.pdf]

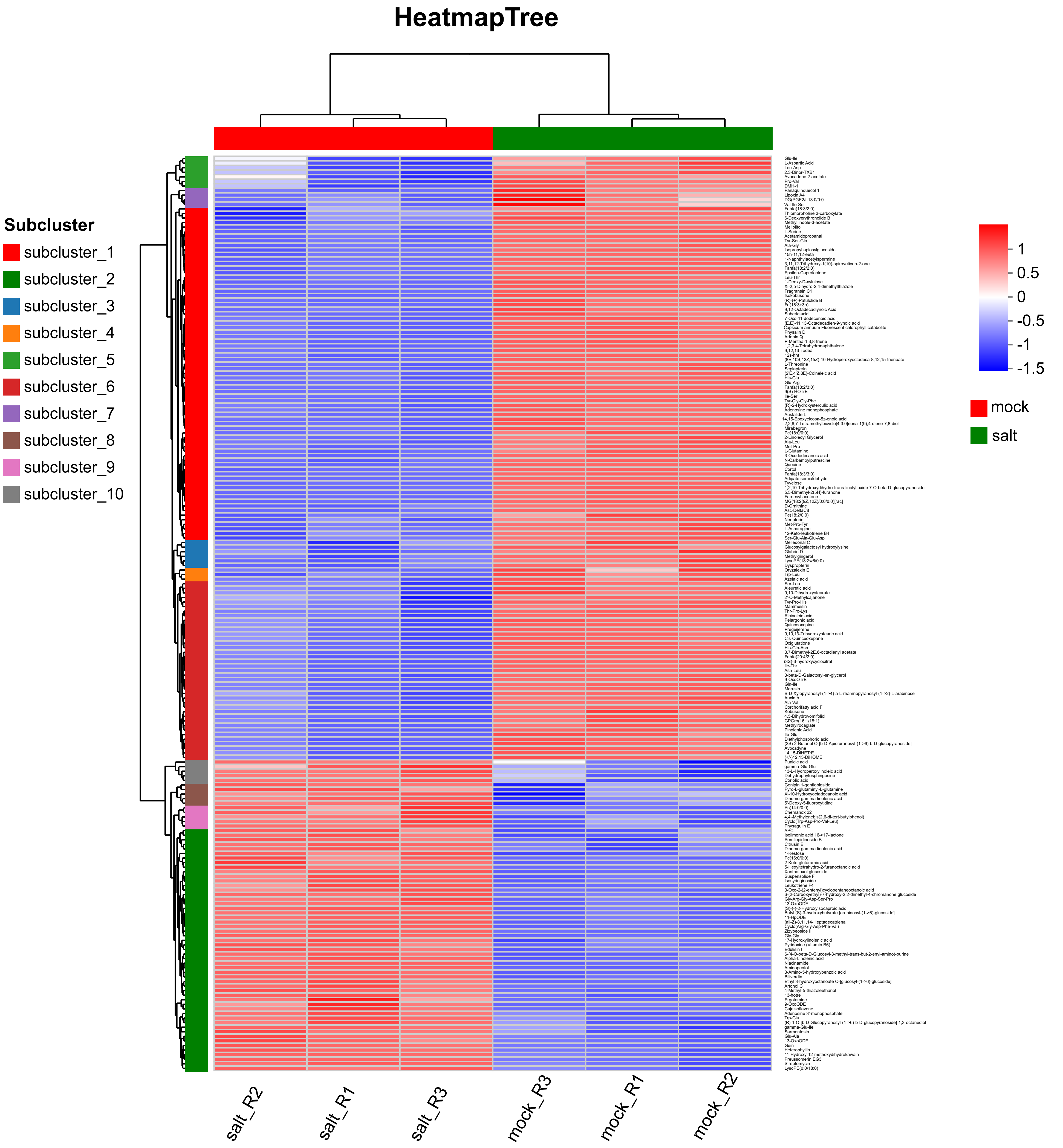

Figure S2: Metabolome analyses reveal changes in cotton in response to salt stress during post-germination stage.
