# Supplementary material for: Integrated Transcriptomic and Metabolomic Analyses Reveal Key Responses of Cotton to Salt Stress Post-Germination
Source: Curr Issues Mol Biol. 2025 Nov 15;47(11):951. doi: 10.3390/cimb47110951 (PMC12651791; doi:10.3390/cimb47110951)
Supplement: Supplementary file 1 [file cimb-47-00951-s001.zip › Figure S3.pdf]

A

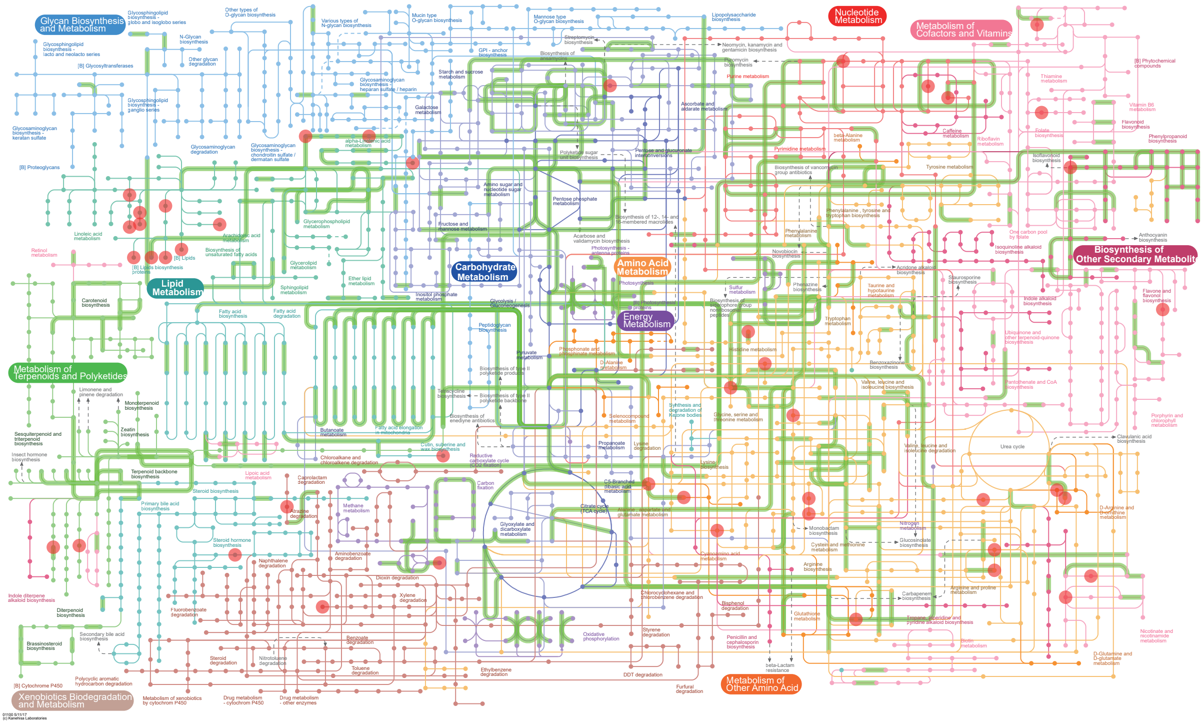

B

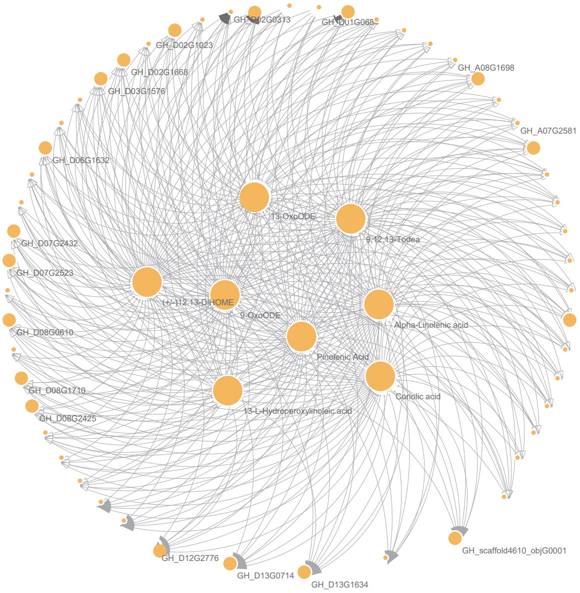

Figure S3: Integration analysis of DEGs and DEMs; (A) iPath analysis of DEGs and DEMs; (B) Connection network between eight metabolites in the top 10 DEMs and screened DEGs (log2FC > 1, p < 0.05, and FPKM > 10).
